# Has the COVID-19 pandemic strengthened confidence in managing the climate crisis? Transfer of efficacy beliefs after experiencing lockdowns in Switzerland and Austria

**DOI:** 10.3389/fpsyg.2022.892735

**Published:** 2022-10-10

**Authors:** Stephanie Moser, Sebastian Seebauer

**Affiliations:** ^1^Centre for Development and Environment, University of Bern, Bern, Switzerland; ^2^Joanneum Research Forschungsgesellschaft mbh, Graz, Austria

**Keywords:** self-efficacy, participative efficacy, collective efficacy, efficacy affect, climate change mitigation, positive spillover, similarity

## Abstract

In the spring of 2020, countries introduced lockdowns as radical measures to deal with the first wave of the COVID-19 pandemic, which led to strong disruptions of people's everyday lives. Such drastic collective measures had previously seemed inconceivable in relation to other urgent crises, such as the climate crisis. In this paper, we ask how individual, participatory, and collective efficacy beliefs in dealing with the COVID-19 pandemic transferred to efficacy beliefs regarding the climate crisis. We present comparative results from two surveys: Study 1 assesses efficacy beliefs among German-speaking Swiss residents (*n* = 1,016), shortly after lockdown measures were relaxed. Study 2 compares changes in efficacy beliefs among Austrian high school students (*n* = 113) before and after the lockdown. In Study 1, climate-related self- and participatory efficacy are enhanced by the corresponding COVID-19-related beliefs. Climate-related efficacy beliefs mediate the effect of COVID-related counterparts on climate-friendly behavior and policy support. Study 2 shows that COVID-19-related efficacy beliefs are transferred to climate-related counterparts over time, and that the transfer of participatory efficacy is moderated by perceived similarity of the two crises. Experiencing successful individual and collective action during the COVID-19 pandemic seems to inspire confidence in dealing with climate change. Underlying processes (direct transfer, consistency, awareness-raising, learning) are discussed.

## Introduction

Curbing climate change is one of the greatest challenges ever faced by humanity. Under the Paris Agreement, the international community has committed itself to limiting global warming to 1.5°C (United Nations, 2016). This means that greenhouse gas (GHG) emissions must be reduced to a net-zero level by 2050 at the latest (IPCC, 2018). The attention to climate change temporarily dropped from public interest in the spring of 2020, however, as the world community was suddenly confronted with another global crisis, the rapidly spreading COVID-19 pandemic. Many governments responded with drastic measures in the form of lockdowns that greatly disrupted public life for a period of time. In this first phase of the COVID-19 pandemic, radical and comprehensive collective crisis management measures were passed, as had previously been demanded in vain in the face of the climate crisis (Reese et al., 2020). Due to pandemic restrictions, people were forced to cut back on consumption and mobility, which had a significant impact on individual and global greenhouse gas emissions (Forster et al., 2020; Le Quéré et al., 2020; United Nations Environment Programme, 2020). During the national lockdowns, people could thus experience how a life with a small GHG-footprint might feel, and what positive effects this could have on nature and human wellbeing (Garrido-Cumbrera et al., 2021). At the same time, this first wave of the COVID-19 pandemic was accompanied by a collective sense of solidarity as well as high public approval of the radical governmental measures taken (Austrian Corona Panel Project, 2020; Sotomo, 2020).

In this first wave of the COVID-19 pandemic, various voices expressed the (hopeful) expectation that experiencing these drastic lockdown measures in everyday life might open a window of opportunity for moving forward with stringent climate action (Reese et al., 2020; Lehmann et al., 2021). The first wave of the COVID-19 pandemic, during which this paper emerged, represented an unexpected real-world opportunity to gain a better understanding of the extent to which, and mechanisms by which, learning from one crisis to deal with another might take place. We wondered whether people's experiences of coping with the COVID-19 pandemic formed corresponding efficacy beliefs that might transfer to efficacy beliefs and behaviors regarding the climate crisis. In this paper, we report the results of two surveys in Switzerland and Austria that were conducted after the national lockdowns ended in the late spring of 2020. Our results suggest that COVID-19-related efficacy beliefs inform the corresponding climate-related efficacy beliefs, even when controlling for climate-related self-identity or the stability of climate-related efficacy beliefs over time.

## Efficacy beliefs as antecedents of climate-friendly private and public behavior

Efficacy beliefs have been shown to be an important predictor of climate-friendly behavior in previous research. Based on social cognitive theory (Bandura, 1997, 2006), different types of efficacy beliefs have been explored in the context of pro-environmental and climate-friendly behavior: self-, collective and participatory efficacy, and efficacy affect.

*Self-efficacy* refers to an individual's belief in being capable of performing a certain action (behavioral self-efficacy; Bandura, 1997), or achieving a certain goal (goal-oriented self-efficacy; Hamann and Reese, 2020), sometimes also referred to as response self-efficacy (Bostrom et al., 2019; Brügger et al., 2020). In environmental psychology, goal-oriented self-efficacy is understood as “an individual's perception of his or her ability to effect positive change regarding the environment” (Sawitri et al., 2015, p. 30). Self-efficacy beliefs thus encourage people to adopt an active, problem-oriented, that is to say, mitigating role in the face of personal or social crises (Homburg and Stolberg, 2006). Several empirical studies underpin the relevance of self-efficacy beliefs for pro-environmental and climate-friendly consumption behavior (known as private sphere behavior, e.g., Tabernero and Hernández, 2011; Hunter and Röös, 2016; Reese and Junge, 2017; Loy et al., 2020), and for an active role in the climate strike movement (known as activism, e.g., Brügger et al., 2020; Cologna et al., 2021), as well as for the support of climate policies (known as public behavior, e.g., Bostrom et al., 2019).

The climate crisis does not present itself only as an individual task in shaping one's own climate-friendly lifestyle, however, but rather as a comprehensive, global problem that requires collective action by all actors at all levels (Capstick et al., 2014; Amel et al., 2017). Given this collective challenge and the required collective solutions, psychological research has increasingly addressed efficacy beliefs with particular explanatory power for activist behavior or public support of climate policies. *Collective efficacy* is understood as the belief that a collective or a group can achieve certain goals and thus contribute to crisis management (Bandura, 1997; Fritsche et al., 2018; Reese et al., 2020). Various studies show empirically that collective efficacy beliefs are associated with both private behavior and public behavior (e.g., Rees and Bamberg, 2014; Barth et al., 2016; Reese and Junge, 2017; Sabherwal et al., 2021). In some cases, self-efficacy is found to be a better predictor of private-sphere behavior and collective efficacy a better predictor of public behavior (Jugert et al., 2016; Hamann and Reese, 2020).

Even free-riders may hold strong collective efficacy beliefs, however, and may presume that others will reach for the common goals without themselves taking an active part (Hamann et al., 2021). *Participatory efficacy*, therefore, encompasses the belief that one's own contribution makes a significant difference to reaching the collective goals (van Zomeren et al., 2013). Participatory efficacy links self- and collective efficacy by focusing on one's own agency within the framework of the collective (Meijers et al., 2021). Various empirical studies report that participatory efficacy has better predictive power than collective efficacy (van Zomeren et al., 2013, 2018; Bamberg et al., 2015; van den Broek et al., 2019).

Efficacy beliefs have not just a cognitive, but also an affective component. *Efficacy affect* comprises emotions toward anticipated future outcomes, just as efficacy beliefs are directed to a certain action or goal (Geiger et al., 2021c). Positive affect, such as hope or enthusiasm, reflects an emotional state of optimistic expectations about the future. Conversely, negative affect, such as frustration or helplessness, signals futility and lack of confidence in attaining a desired outcome. Emotional states are a source of self-efficacy and other efficacy beliefs (Bandura, 1977; Hamann et al., 2021); for instance, being positively moved is related to collective efficacy (Landmann and Rohmann, 2020). Positive affect predicts pro-environmental behavior (Hamann and Reese, 2020; Hamann et al., 2021). Hostile emotions, such as anger and outrage, instigate collective action when protesting against personal deprivation and injustice (Bamberg et al., 2015); by contrast, pro-environmental activists, who are rarely disadvantaged in their personal livelihood by the topics they act on, are motivated by feelings of hope or of being moved (Landmann and Rohmann, 2020; Geiger et al., 2021c).

Apart from their direct predictive power, efficacy beliefs are assumed to function as a mechanism when learning from one context to deal with another (Nash et al., 2017). Performance accomplishments, that is to say, previous experiences of mastering challenges, are a source of self-efficacy (Bandura, 1977). In this sense, successfully employing personal capabilities when coping with the COVID-19 crisis can be expected to influence efficacy beliefs toward other crises. Efficacy beliefs are discussed as a mechanism for positive spillover, which is the phenomenon when performing an initial pro-environmental behavior increases the likelihood of performing other pro-environmental actions later or in a different context (Nilsson et al., 2017; Carrico, 2021). Accomplishing the initial behavior supposedly reinforces corresponding efficacy beliefs, which then promote other pro-environmental actions (Thøgersen and Crompton, 2009). The role of efficacy beliefs as spillover mechanism is, however, debated (Lauren et al., 2016; Egner and Klöckner, 2021). In direct comparison, ecological self-identity emerges as a more relevant mediator for spillover than efficacy beliefs (Lauren et al., 2019). Spillover is more likely between behaviors from a similar functional category or directed toward a similar goal (Lauren et al., 2016; Nash et al., 2017; Höchli et al., 2019). If similarity moderates the transfer between behaviors, it seems plausible that similarity also guides the transfer of efficacy beliefs. All the same, recent meta-analyses caution against expecting substantial spillover effects in behaviors (Maki et al., 2019; Geiger et al., 2021a).

## Perceived similarity and transfer between the COVID-19 pandemic and the climate crisis

The notion of learning from COVID-19 for the climate crisis had already been put forward at the beginning of the pandemic (Reese et al., 2020; Lehmann et al., 2021). Several studies examine similarities in the public perception of the COVID-19 crisis and the climate crisis: Bostrom et al. (2020) find that U.S. citizens perceive both crises as highly threatening and hardly controllable. The climate crisis is considered to be better understood, however, and people think that they can contribute more to mitigating the COVID-19 crisis, and feel a greater moral responsibility to do so, compared to the climate crisis. In Geiger et al. (2021b), U.S. citizens report more perceived similarities between the two crises than perceived differences. Emotional evaluation of the crises had greater explanatory power for crisis-related behaviors in the case of the climate crisis than in the case of the COVID-19 crisis; however, both studies remain at a descriptive level and do not investigate possible transfer processes between the two crises.

To the best of our knowledge, only a few studies compare the effect of efficacy beliefs on climate- and pandemic-related behavior. Meijers et al. (2021) find in a Dutch sample that COVID-19-related participatory efficacy predicts pandemic-mitigating behavior, and climate-related participatory efficacy predicts climate change-mitigating behavior; however, this study does not test for transfer processes between the two crises. By contrast, Lucarelli et al. (2020) find in an Italian student sample that perceptions of existing interdependencies between the COVID-19 crisis and the climate crisis strengthen the link between perceived behavioral control (a construct related to self-efficacy; Ajzen, 2002) and climate-related behavioral intentions, as well as corresponding private behavioral implementation.

## Present research

This paper reports the results of two studies that were conducted as an *ad hoc* cooperation at the beginning of the pandemic lockdowns in spring 2020 in two different countries. Study 1 was part of a large-scale survey on the experience and reactions during the lockdown in Switzerland. Study 2 extended an already planned longitudinal study on climate attitudes and behaviors among high school students in Austria. Both studies apply identical questionnaire items and complement each other in terms of survey design, sample size, and representativeness. Both studies should be considered exploratory as they reacted to the novelty and research opportunity of the pandemic situation in spring 2020 and the short timeframe when lockdowns were lifted; therefore, the studies were not preregistered.

Both studies address two overarching research questions. First, we analyze whether COVID-19-related efficacy beliefs directly inform corresponding climate-related efficacy beliefs; in other words, we test for their positive association. Therein, we take up the argumentation that the pandemic experience demonstrated individual and collective capabilities that may transfer to coping with the climate crisis (Lauren et al., 2019; Reese et al., 2020; Lehmann et al., 2021). Second, we explore mechanisms, conditions, and behavioral effects related with the transfer of efficacy beliefs. Concordant with the perspective on efficacy beliefs as spillover mechanism, Study 1 tests whether the effect of COVID-19-related efficacy beliefs on different climate-friendly behavioral responses is mediated via the corresponding climate-related efficacy beliefs (Lauren et al., 2016). In line with the perspective that spillover is more likely to occur between similar contexts, Study 2 tests whether the effect of COVID-19- on climate-related efficacy beliefs is moderated by the perceived similarity between the COVID-19 and the climate crisis (Lauren et al., 2016; Maki et al., 2019).

## Study 1

**Figure 1:**
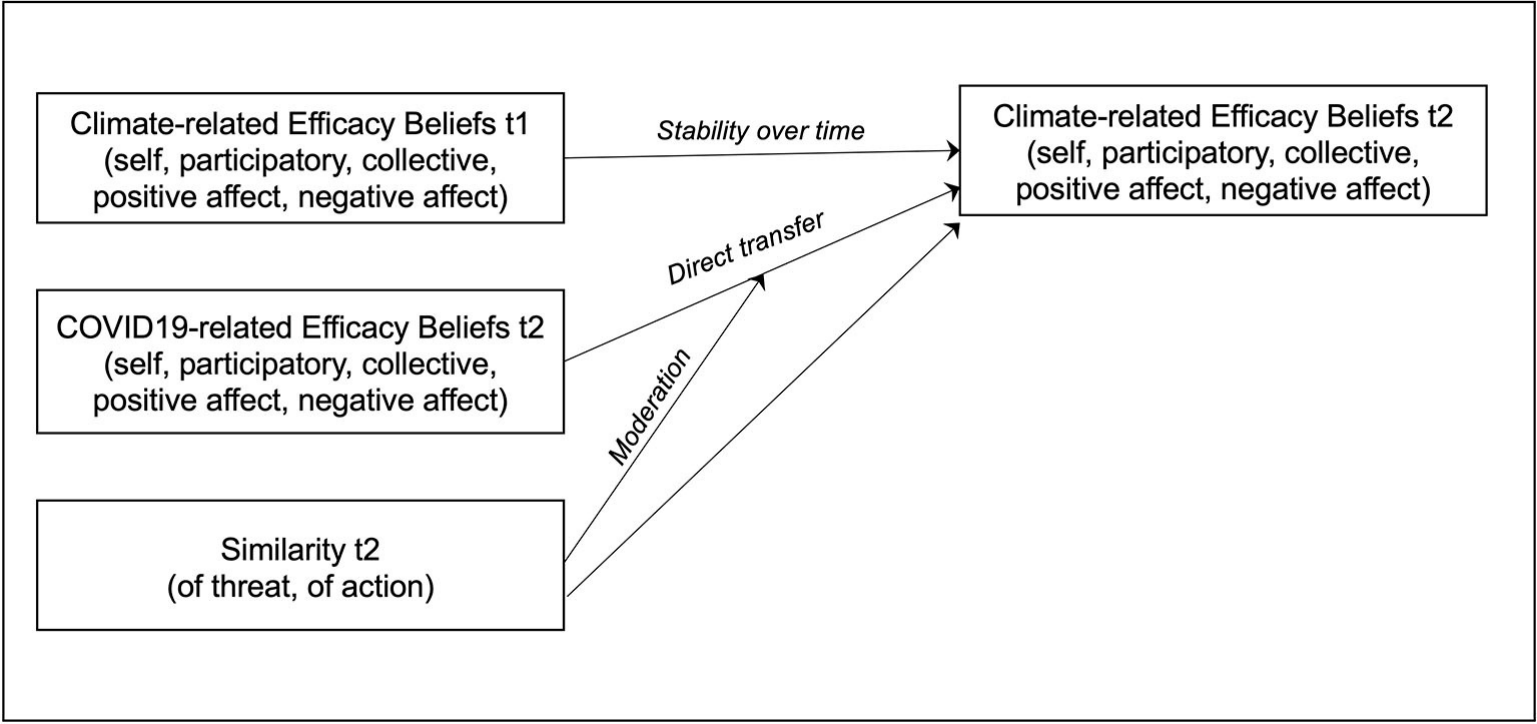
Mediation model of Study 1 assuming an indirect effect of COVID-19-related efficacy beliefs on climate-friendly behavioral responses *via* climate-related efficacy beliefs (The labeling of the paths facilitates interpretation of the estimates in Tables 1, 2).

Study 1 tests a mediation model in which COVID-19-related efficacy beliefs are assumed to inform corresponding climate-related efficacy beliefs and thus indirectly affect corresponding climate-friendly behavioral responses, namely individual private-sphere pro-environmental behavior and policy support (see Figure 1). We thus refer to the two overarching research questions, in other words, the assumed positive relationship between COVID-19-related and climate-related efficacy beliefs, and the assumed indirect effect of COVID-19-related efficacy beliefs on climate-friendly private behavior and policy support. Previous studies have shown that both, self-identity and self-efficacy have the potential to promote spillover effects (Lauren et al., 2016, 2019). We therefore included self-identity to test whether self-efficacy leads to transfer effects even when self-identity is controlled.

### Methods

#### Participants

Study 1 was conducted as a cross-sectional survey from May 07-14, 2020, just as the first lockdown in Switzerland, which had started on March 16, was gradually lifted. In addition to the contents reported in this paper, the survey captured changes in time use, consumption behavior, and wellbeing during the lockdown. A sample of German-speaking Swiss residents aged 18 to 65 was recruited from an existing panel of a survey institute (Intervista), with quotas set by gender, age, and education. Participants were invited to a standardized online questionnaire by the survey institute and were rewarded for completing the survey with the institute's point-based voucher system. The invitation was made by the panelist institute, i.e., the researchers did not have personal or contact information at any time, and the panelist institute did not have access to the participants' responses. The panelists were persons who had in principle given consent to the panelist institute to participate in surveys and they could decline the invitation to this particular study. On the survey landing page, the participants were informed about the purpose and conditions (e.g., anonymity, confidentiality, voluntariness, option to cancel or interrupt) of the study and had to explicitly state their informed consent before starting the survey. In total, 1,176 participants were invited, of whom 1,051 (89.4%) completed the survey. Thirty-five “speeders” were excluded who completed the questionnaire in <10 min (average completion time: 21 min). This yielded a sample of *n* = 1,016 participants for analysis (net sample = 86.4%).

Participants were not required to respond to all items, so the number of missing values varies between items. All Study 1 results are calculated applying listwise exclusion of missing values which reduces the sample size marginally to *n* = 985–994. The sex ratio of the sample was 50%, as targeted, and the mean age was 43.07 years (*SD* = 13.26). Household size averaged 2.43 persons (*SD* = 1.21, with a *min* = 1 and *max* = 8). 1.1% had not concluded compulsory education, 4.6% reported compulsory education as their highest level of education, 54.8% had a vocational apprenticeship or vocational school diploma, 10.3% had a high school degree (Maturität), and 27.9% had a university degree. These sociodemographic characteristics indicate to a high diversity of the sample that is not equivalent but comparable to the Swiss resident population in terms of age, gender, family size, and educational degree (Federal Statistical Office, n.d; Federal Statistical Office., 2021).

#### Procedure and materials

For detailed wording (translated from German), descriptive statistics, and scale reliabilities of all items please refer to Supplementary Table S1. Efficacy beliefs were assessed as two distinct factors, referring to agency as an individual (*self-efficacy*, two items; e.g., “I am confident that I can do something for climate protection on my own”), and as a member of a group (*participatory efficacy*, two items; e.g., “I am capable of making a small but important contribution to improving climate protection together with others”). Item wordings were informed by previous studies (Bamberg et al., 2015; Lauren et al., 2016; Hamann et al., 2021). The wording was adjusted to create parallel versions of these items referring to either climate protection or tackling the COVID-19 crisis. Responses were given on a rating scale from “strongly agree” (5) to “strongly disagree” (1). Cronbach's alpha for these four factors ranged from α = 0.75 to α = 0.86, indicating good scale reliability (Bortz and Döring, 2006). Items assigned to the respective factors were aggregated to mean factor indices to correct for measurement error of single items; thus, the unstandardized regression coefficients of factors reported in the Results section of Study 1 below use the same five-step scale as the single items. Intercorrelations of the different efficacy beliefs are between *r* = 0.29 and *r* = 0.60 (see Supplementary Table S2).

Climate change-related behavior responses (the outcome variables) were assessed as two different behavioral responses: considering the COVID-19 crisis as a window of opportunity for radical climate policies (*policy support*, four items based on own wording, α = 0.82; e.g., “To protect the climate, I would welcome profound governmental measures similar to those taken to combat the COVID-19 pandemic”); environmentally friendly private behavior during the COVID-19 lockdown (seven items based on own wording, applying an answer scale from (5) “fully true” to (1) “not true at all,” α = 0.71; e.g., “I have repaired items that were broken”). For both factors, again, mean factor indices were formed for the further analyses.

For measuring *climate-related self-identity* (the covariate), three items were slightly adapted from Van der Werff et al. (2014; e.g., “I am the type of person who acts in a climate-friendly way”); these items used the “strongly agree” (5) to “strongly disagree” (1) rating scale and were aggregated to a mean index (α = 0.92). Items assessing climate-related efficacy beliefs, public behavior, and climate-related self-identity were presented in the same block in randomized order. Items on COVID-19-related efficacy and private behavior were each presented in separate blocks.

#### Analytical approach

Study 1 tests four versions of the mediation model in Figure 1, which assumes that the predictor COVID-19-related efficacy indirectly affects the outcome variable climate-relevant private behavior and policy support (the latter as a specific case of public behavior) *via* the mediator variable climate-related efficacy while controlling for the covariate climate-related self-identity. We calculated four regression models, each combining self- or participatory efficacy with private behavior and policy support. We calculated, following Hayes (2018) and Hayes and Rockwood (2017) the direct, the indirect, and the total effects of efficacy beliefs on behavioral responses using the PROCESS program for SPSS. We first regressed the mediator variable on the predictor and the covariate, and second, we regressed the outcome variable on the predictor, the mediator, and the covariate. The indirect effect of the predictor *via* a mediator on the outcome variable results from the product of the path estimates *a* and *b* (cp. Figure 1). All results are tested against *p* < 0.05, *p* < 0.01, and *p* < 0.001 significance levels. Modified Breusch-Pagan tests for heteroscedasticity were significant for three of the four models, we thus applied robust standard errors (HC3 Davidson McKinnon) and covariance matrix estimators. Moreover, we report 10,000 bootstrapped confidence intervals.

### Results

We first test for the assumption of positive associations between COVID-19-related and climate-related efficacy beliefs. As shown in the leftmost part of Table 1, the mediator variable climate-related self-efficacy is statistically significantly predicted by the predictor COVID-19-related self-efficacy (*a* = 0.22^***^), and the covariate climate-related self-identity (*f* = 0.65^***^, *R*^2^ = 0.48). Table 2 reveals a comparable picture: The mediator climate-related participatory efficacy is predicted by COVID-19-related participatory efficacy (*a* = 0.31^***^), as well as climate-related self-identity (*f* = 0.60^***^, *R*^2^ = 0.40). Our assumption of a positive relationship between the efficacy beliefs of both crises is thus confirmed for self-efficacy as well as for participatory efficacy.

**Table 1:** Model coefficients for the mediation model assuming an indirect effect of COVID-19-related self-efficacy on behavioral responses *via* climate-related self-efficacy, with self-identity as covariate.

	**Self-efficacy (Climate)**					**Policy support**					**Private behavior**				
**Predictor**	**Parameter**	**Estimate**	***SE***	***p***	**95% C.I**.	**Parameter**	**Estimate**	***SE***	***p***	**95% C.I**.	**Parameter**	**Estimate**	***SE***	***p***	**95% C.I**.
					**(LL, UL)**					**(LL, UL)**					**(LL, UL)**
Constant	*i_*M*_*	0.70	0.12	0.000	(0.46, 0.94)	iy	0.86	0.15	0.000	(0.57, 1.14)	*i_*y*_*	1.64	0.12	0.000	(1.41, 1.88)
Self-efficacy (COVID-19)	*a*	0.22	0.03	0.000	(0.17, 0.27)	c'	0.10	0.03	0.001	(0.04, 0.17)	*c'*	0.13	0.03	0.000	(0.08, 0.18)
Self-efficacy (Climate)		-	-	-	-	b	0.22	0.04	0.000	(0.13, 0.30)	*b*	0.07	0.04	0.051	(−0.00, 0.15)
Self-identity (Climate)	*f*	0.65	0.03	0.000	(0.60, 0.71)	g	0.41	0.04	0.000	(0.32, 0.49)	*g*	0.16	0.04	0.000	(0.09, 0.23)
	*R^2^* = 0.48					*R^2^* = 0.31					*R^2^* = 0.11				
	*F*(2, 988) = 401.12, *p* < 0.001					*F*(3, 987) = 136.12, *p* < 0.001					*F*(3, 990) = 39.73, *p* < 0.001				
	*N* = 991					*N* = 991					*N* = 994				

Parameters correspond to the paths in Figure 1. Estimate, unstandardized regression coefficient; SE, robust standard error (HC3), 95% C.I. (LL, UL) = 95% bootstrap confidence interval (lower level, upper level).

**Table 2:** Model coefficients for the mediation model assuming an indirect effect of COVID-19-related participatory efficacy on behavioral responses *via* climate-related participatory efficacy, with self-identity as covariate.

	**Participatory efficacy (Climate)**					**Policy support**					**Private behavior**				
**Predictor**	**Parameter**	**Estimate**	***SE***	***p***	**95% C.I**.	**Parameter**	**Estimate**	***SE***	***p***	**95% C.I**.	**Parameter**	**Estimate**	***SE***	***p***	**95% C.I**.
					**(LL, UL)**					**(LL, UL)**					**(LL, UL)**
Constant	*i_*M*_*	−0.18	0.12	0.127	(−0.42, 0.05)	iy	1.06	0.14	0.000	(0.79, 1.33)	*i_*y*_*	1.67	0.12	0.000	(1.44, 1.90)
Participatory efficacy (COVID-19)	*a*	0.31	0.03	0.000	(0.25, 0.37)	c'	0.07	0.03	0.011	(0.02, 0.13)	*c'*	0.13	0.03	0.000	(0.08, 0.18)
Participatory efficacy (Climate)		-	-	-	-	b	0.24	0.03	0.000	(0.18, 0.31)	*b*	0.08	0.03	0.008	(0.02, 0.14)
Self-identity (Climate)	*f*	0.60	0.03	0.000	(0.54, 0.67)	g	0.40	0.04	0.000	(0.32, 0.47)	*g*	0.16	0.03	0.000	(0.09, 0.22)
	*R^2^* = 0.40					*R^2^* = 0.33					*R^2^* = 0.12				
	*F*(2, 982) = 368.35, *p* < 0.001					*F*(3, 981) = 158.73, *p* < 0.001					*F*(3, 983) = 43.12, *p* < 0.001				
	*N* = 985					*N* = 985					*N* = 987				

Parameters correspond to the paths in Figure 1. Estimate, unstandardized regression coefficient. SE, robust standard error (HC3), 95% C.I. (LL, UL) = 95% bootstrap confidence interval (lower level, upper level).

Second, we test for the hypothesized indirect effect of COVID-19-related efficacy beliefs on climate-friendly behavioral responses *via* climate-related efficacy beliefs. The estimates in the medium part of Table 1 show that policy support is predicted by statistically significant direct effects of the predictor COVID-19-related self-efficacy (*c'* = 0.10^**^), the mediator climate-related self-efficacy (*b* = 0.22^***^), and the covariate self-identity (*g* = 0.41^***^, *R*^2^ = 0.31). The indirect effect of COVID-19-related self-efficacy *via* climate-related self-efficacy results from the product of the parameters a^*^b = 0.22^*^0.22 = 0.05 and is statistically significant (*SE(HC3)* = 0.01, *Z* = 4.30, *p* = 0.000, 95% C.I. [0.03, 0.07]) according to Sobel's test (Hayes, 2018). The total effect of COVID-19-related self-efficacy on policy support behavior amounts to *c* = *c'*+a^*^b = 0.15 (*p* = 0.000, 95% C.I. [0.09, 0.21]). Thus, when controlling for climate-related self-identity, the influence of COVID-19-related self-efficacy on policy support is partially mediated by climate-related self-efficacy.

The center part of Table 2 shows the same mediation analysis for participatory efficacy. We find a statistically significant indirect effect of COVID-19-related participatory efficacy via climate-related participatory efficacy on policy support (a^*^b = 0.31^*^0.24 = 0.07, *SE(HC3)* = 0.01, *Z* = 5.91, *p* = 0.000, 95% C.I. [0.05, 0.10]). The total effect of COVID-19-related participatory efficacy on public support amounts to *c* = *c'*+a^*^b = 0.15 (*p* = 0.000, 95% C.I. [0.09, 0.21]). Thus, COVID-19-related participatory efficacy also shows partial mediation *via* climate-related participatory efficacy on policy support while controlling for climate-related self-identity.

Finally, we repeat the mediation analysis for climate-friendly private behavior during the COVID-19 lockdown. The first regression step involving efficacy beliefs and self-identity is identical to the above results on policy support. As can be seen from the right-hand part of Table 1, in the second regression step, the effect of the mediator climate-related self-efficacy on private behavior does not reach statistical significance (*b* = 0.07^n.s.^). Consequently, the indirect effect of COVID-19-related self-efficacy on private behavior does not reach significance either (a^*^b = 0.22^*^0.07 = 0.02, *SE(HC3)* = 0.01, *Z* = 1.90, *p* = 0.058, 95% C.I. [0.00, 0.03]). The total effect is significant (*c* = *c'*+a^*^b = 0.15, *p* = 0.000, 95% C.I. [0.09, 0.20]) due to a significant direct effect of COVID-19-related self-efficacy (*c'* = 0.13^***^). Thus, environmentally friendly everyday activities during the lockdown cannot be attributed to an increased climate-related self-efficacy. By contrast, the indirect effect of COVID-19-related participatory efficacy *via* climate-related participatory efficacy on private behavior proves statistically significant (a^*^b = 0.31^*^0.08 = 0.03, *SE(HC3)* = 0.01, Z = 2.55, *p* = 0.011, 95% C.I. [0.01, 0.05]). The total effect of COVID-19-related participatory efficacy on private behavior is also significant (*c* = *c'*+a^*^b = 0.16, *p* = 0.000, 95% C.I. [0.11, 0.21]), as is the direct effect of COVID-19-related participatory efficacy on private behavior (c' = 0.13^***^). This partial mediation suggests that people who were convinced of their personal contribution in collectively mastering the COVID-19 crisis also carried out more environmentally friendly activities in their private lives during the lockdown.

### Discussion

The results of Study 1 confirm the hypothesized positive associations between COVID-19-related and climate-related beliefs for self- and participatory efficacy. Both COVID-19-related self- and participatory efficacy indirectly translate (*via* partial mediation by climate-related efficacy) into increased support for radical and comprehensive climate-protective policies similar to those used to combat the pandemic. Indirect transfer *via* partial mediation also applies to the effect of COVID-19-related participatory efficacy on private environmentally friendly action during the pandemic lockdown. Only for self-efficacy and private behavior can the assumed mediating effect not be confirmed when controlling for self-identity. Thanks to a comment from an anonymous reviewer, we re-analyzed our models without the covariate self-identity to exclude a possible confounding effect of self-identity. The results can be found in Supplementary Tables S3, S4. Without the covariate self-identity, all four mediation analyses proved to be statistically significant, i.e., also the partial mediation effect of self-efficacy on private behavior (with an indirect effect of a^*^b = 0.33^*^0.17 = 0.06, *SE(HC3)* = 0.01, *Z* = 5.31, *p* = 0.000, 95% C.I. [0.04, 0.08], and a total effect of *c* = *c'*+a^*^b = 0.18 (*p* = 0.000, 95% C.I. [0.13, 0.23]). This re-analysis suggests that self-identity acts as an important confounder, at least in this latter case, and that it important to consider self-identity also in corresponding future analyses.

The Study 1 results are, however, based on cross-sectional data; thus, the causality of the relationships within the tested mediation model relies on theoretical assumptions, and the correlational data would also allow for the opposite causal direction of climate-related on COVID-19-related efficacy beliefs. Moreover, Study 1 does not make any statements about conditions under which the transfer of efficacy beliefs between both crises becomes more likely. Both of these limitations are addressed in Study 2.

Study 1 relies on self-reported, not observed behavioral measures that only capture a limited range of climate-related actions Swiss citizens may take. Private behavior as measured in Study 1 encompasses rather general ecological domains which the lockdown provided an opportunity to discover (e.g., repairing and decluttering personal belongings). Private behavior does not include carbon-intensive behaviors because these were restricted for all during the lockdown (e.g., everyday transport, holiday travel) or were unlikely to shift from established patterns (e.g., domestic heating). Support of climate policy is a very specific case of public behavior and does not include political activism. Partial congruence between general efficacy beliefs related to climate protection and selected specific behaviors could be a reason for statistically weak effects.

## Study 2

**Figure 2:**
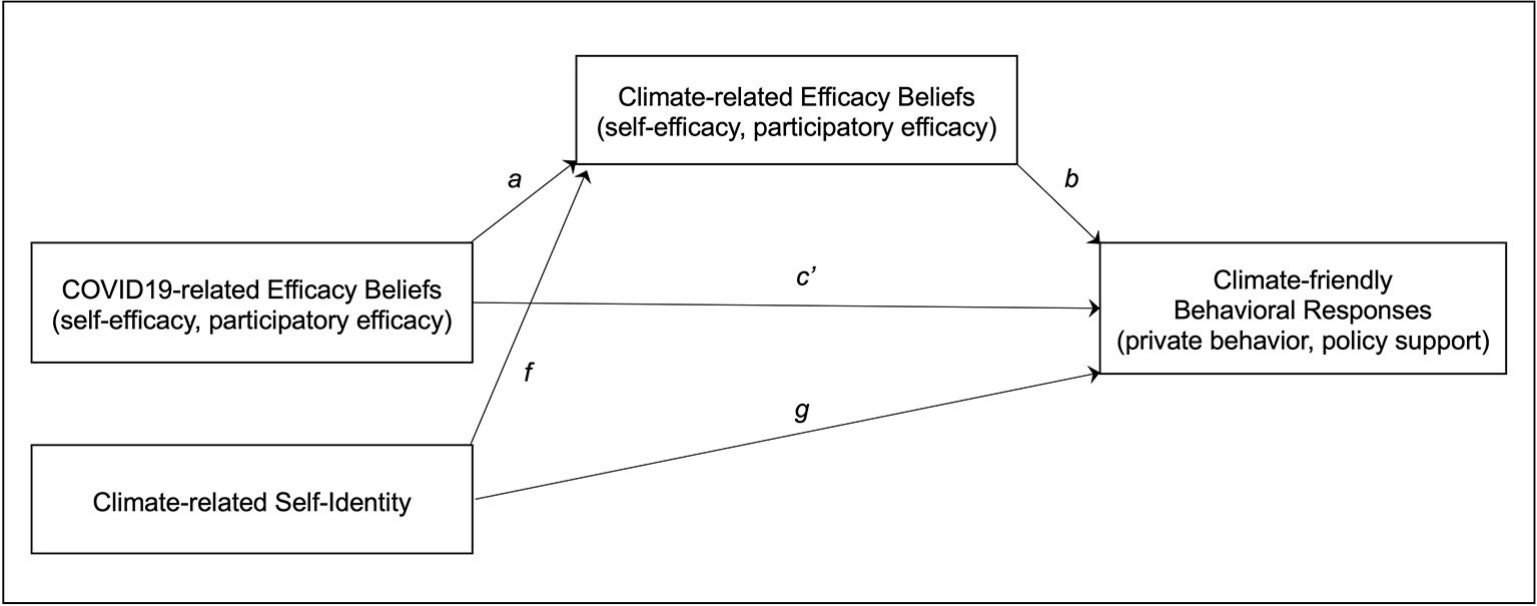
Moderation model of Study 2 assuming that the transfer of efficacy beliefs between the COVID-19 and the climate crises is more likely if the two crises are perceived as similar.

Study 2 tests a moderation model in which the effect of COVID-19-related efficacy beliefs on climate-related efficacy beliefs is assumed to depend on the perceived similarity of the two crises (see Figure 2). We thus refer to our two overarching research questions, that is to say, the assumed positive relationship between COVID-19-related and climate-related efficacy beliefs, and that transfer of efficacy beliefs is more likely between contexts perceived as similar. We test whether the relationships for self-efficacy and participatory efficacy observed in Study 1 can be replicated with longitudinal data. In addition, we test the proposed model for collective efficacy, and positive and negative efficacy affect.

### Methods

#### Participants

Study 2 was implemented as a longitudinal survey among Austrian high school students before and after the COVID-19 school closures in Spring 2020. In February to March 2020, just before the national lockdown started, students in their final high school year (12^th^ or 13^th^ year of formal education) were surveyed as part of a research project on climate attitudes and behaviors (first survey wave, t1). In May 2020, after quarantine rules had been relaxed and teaching in classrooms recommenced, these students were approached again (second survey wave, t2).

At t1, students in four general and two vocational secondary schools in the Austrian province of Styria completed standardized electronic questionnaires in the classroom during school hours. A researcher was present on-site for oversight and clarification. At t2, those students who had provided valid contact data at t1, received an email invitation to an online questionnaire, followed by up to two reminder emails. In total, 300 students participated at t1; 231 students gave contact data at t1; 113 students participated at t2, amounting to a response rate of 49% and yielding a longitudinal sample for analysis of *n* = 113 cases. Respondents were aged 16 to 20 years (mean = 17.9, *SD* = 0.79) and 53.1% female. The age distribution remained fairly constant from t1 to t2; however, male students and students from vocational schools were less represented at t2 (Supplementary Table S5 details sample dropout from withholding contact data or panel attrition). The data do not include any missing values in any variables.

Study 2 was conducted in line with the ethics guidelines of the authors' home institutions and of the provincial school boards in Austria. In Study 2, 65.5% of the subjects were of legal age of 18 years; by Austrian law, youths of 14 years are legally competent for minor commercial activities including the use of online services. The Study 2 questionnaire included an introduction stating the study purpose and ensuring confidentiality of information (in particular with regards to teachers, parents or peers), and a detailed data protection statement. Survey participation was voluntary; even in the classroom at t1, students could cancel the survey and delete all responses at any point of the questionnaire.

#### Procedure and materials

For detailed wording (translated from German), descriptive statistics, and scale reliabilities of all items please refer to Supplementary Table S6. Efficacy beliefs were assessed as three distinct factors, referring to the agency as an individual (*self-efficacy*, three items, two of which were phrased identically as in Study 1), as a member of a group (*participatory efficacy*, three items, two of which were phrased as in Study 1) and as a whole group (*collective efficacy*, three items; e.g., “Through collective efforts of myself and other people, we can achieve progress in climate protection”; van Zomeren et al., 2013; Bamberg et al., 2015; Lauren et al., 2016; Hamann et al., 2021). Emotions associated with efficacy were measured as *positive efficacy affect* (feeling hopeful or motivated, two items; Hamann and Reese, 2020) and as *negative efficacy affect* (feeling helpless or frustrated, two items; Geiger et al., 2021c). Efficacy items were phrased identically for climate and COVID-19, apart from specifying the goal of either climate protection or tackling the pandemic. Responses were given on a rating scale from “fully agree” (5) to “fully disagree” (1). Items on participatory and collective efficacy referred to the ingroup of other young people regarding the climate crisis, and to the ingroup of other people regarding the COVID-19 crisis. Climate-related efficacy beliefs were measured at t1 and t2. COVID-19-related efficacy beliefs were measured only at t2, because at t1 COVID-19 had not yet been an issue neither to us researchers nor to the students.

For assessing perceived similarity between the two crises, respondents were asked at t2 to compare both crises on ten attributes using a five-step bipolar rating scale from “very similar” (+2) to “very dissimilar” (-2). The attributes reflected hazard characteristics identified in the psychometric risk paradigm (originally by Fischhoff et al., 1978) such as dread potential, scientific knowledge, or control over the hazard. *Post hoc* principal component analysis suggested aggregating seven of these attributes to two factors (Supplementary Table S7): *similarity of threat*, considering COVID-19 and climate as pressing and hard-to-manage crises that both require precautionary and collaborative efforts (four items) and *similarity of action*, considering both COVID-19 and climate as crises that can be effectively managed by the state in the short term (three items).

Items were presented in mixed order in the questionnaire to avoid artificial inflation of intra-scale correlations. As in Study 1, items assigned to the respective factors were aggregated to mean factor indices; thus, the unstandardized regression coefficients of factors reported in the Results Section of Study 2 below use the same five-step scale as the single items. Most indices have reliability scores of Cronbach's α > 0.60 or higher, apart from positive efficacy affect and similarity of action (α = 0.55 and α = 0.52); however, all items comply with the common threshold of an item-total correlation *r*_*it*_> 0.30 with their respective index. Short scales consisting of only a few items are generally susceptible to weak reliability (Bortz and Döring, 2006). Mean factor indices of efficacy beliefs intercorrelate moderately at *r* < 0.60, thus indicating satisfactory discriminant validity (Supplementary Table S8). However, self-efficacy and participatory efficacy correlate at *r* = 0.60 to.70, and participatory efficacy and collective efficacy correlate at *r* = 0.70–0.80, which might point to a shared background variable such as general efficacy beliefs or locus of control. Still, for conceptual completeness all efficacy beliefs are analyzed separately.

#### Analytical approach

We calculated ten separate regression models each combining a specific efficacy belief (self, participatory, collective, positive affect, negative affect) with a specific similarity (threat, action). All models have the same structure, regressing climate-related efficacy beliefs at t2 on three predictors (Figure 2): (1) climate-related efficacy beliefs at t1 to control for the autoregressive effect and to show how stable efficacy beliefs are over time; (2) the counterpart COVID-19-related efficacy at t2 to determine its unique additional effect and to show whether efficacy beliefs are directly transferred from COVID-19 to climate; and (3) similarity to show whether the perceived attributes of the COVID-19 crisis change climate-related efficacy. As a transfer of efficacy beliefs is more likely between similar contexts, the models include a COVID-19-related efficacy x similarity interaction term to check for a moderator effect, in other words, whether transfer of efficacy beliefs is more pronounced the more similar both crises are perceived to be. This regression approach serves the dual purpose of showing the stability of climate-related efficacy beliefs, and analyzing how much of the individual variance left unexplained by stability can be traced back to the influence of COVID-19-related efficacy beliefs.

The influence of climate-related efficacy at t1 may be interpreted as a common main effect; however, the influences of COVID-19-related efficacy beliefs and similarity are conditional simple effects because these predictors are also included in the interaction term and are therefore mean-centered for clearer interpretation (Hayes, 2018). Index intercorrelations *r* < 0.66 suggest that there are no issues of multicollinearity among the predictors (Iacobucci et al., 2016). All results are tested against *p* < 0.05 and *p* <0.01 significance levels. Note that we expect positive signs of the regression coefficients in the models on positive as well as negative efficacy affect, as the affective state is transferred from t1 to t2 or from the COVID-19 to the climate crisis regardless of whether the transferred emotions are positive or negative.

### Results

Tables 3, 4 give the regression results; each column reports a separate model that refers in all climate- and COVID-19-related efficacy factors to the same specific efficacy belief (self, participatory, etc.). As an example, we interpret the unstandardized regression coefficients in the model on climate-related self-efficacy and similarity of threat (furthest left column in Table 3): An increase in climate-related self-efficacy at t1 by one step on the five-step response scale leads to an increase in climate-related self-efficacy at t2 by 0.52 steps (*b* = 0.52^**^). This indicates high stability over time. The coefficients of COVID-19-related self-efficacy and similarity of threat have to be read as conditional on the other component of the interaction term: Among those who are average in similarity of threat (because of mean-centering), an increase in COVID-19-related self-efficacy by one step leads to an increase in climate-related self-efficacy at t2 by 0.19 steps (*b* = 0.19^*^). This indicates a direct transfer of efficacy beliefs. Vice versa, among those who are average in COVID-19-related self-efficacy, an increase in similarity of threat by one step leads to an increase in climate-related self-efficacy at t2 by 0.16 steps (*b* = 0.16^*^). This indicates that the COVID-19 crisis raised awareness of the role everyone has to play in reaching a common goal. The interaction term in this example is not statistically significant.

**Table 3:** Model coefficients for moderation models assuming a moderating effect of perceived similarity of threat on the transfer of COVID-19-related efficacy beliefs on climate-related efficacy beliefs.

**Predictor**	**Efficacy (Climate) t2**				
	**Self**	**Participatory**	**Collective**	**Positive affect**	**Negative affect**
Constant	1.86 (0.30)^**^	1.69 (0.25)^**^	1.48 (0.32)^**^	1.50 (0.22)^**^	1.56 (0.22)^**^
Efficacy (Climate) t1	0.52 (0.09)^**^	0.54 (0.07)^**^	0.60 (0.08)^**^	0.53 (0.07)^**^	0.43 (0.07)^**^
Efficacy (COVID-19) t2	0.19 (0.08)^*^	0.10 (0.10)	0.14 (0.11)	0.29 (0.08)^**^	0.24 (0.08)^*^
Similarity of threat t2	0.16 (0.08)^*^	0.31 (0.08)^**^	0.18 (0.09)	0.22 (0.08)^**^	−0.03 (0.10)
Interaction efficacy (COVID-19) × Similarity of threat	−0.00 (0.08)	0.00 (0.11)	0.07 (0.12)	0.06 (0.09)	0.04 (0.10)
*R^2^*	0.42	0.52	0.46	0.52	0.34
*F* (df1 = 4/df2 = 108)	19.33^**^	29.10^**^	23.36^**^	28.53^**^	13.67^**^

* p < 0.05,

** p < 0.01. n = 113. Unstandardized regression coefficients b with standard errors (SE) in parentheses.

**Table 4:** Model coefficients for moderation models assuming a moderating effect of perceived similarity of action on the transfer of COVID-19-related efficacy beliefs on climate-related efficacy beliefs.

**Predictor**	**Efficacy (Climate) t2**				
	**Self**	**Participatory**	**Collective**	**Positive affect**	**Negative affect**
Constant	1.76 (0.30)^**^	1.52 (0.26)^**^	1.44 (0.31)^**^	1.41 (0.22)^**^	1.54 (0.22)^**^
Efficacy (Climate) t1	0.55 (0.09)^**^	0.58 (0.07)^**^	0.61 (0.08)^**^	0.56 (0.07)^**^	0.44 (0.07)^**^
Efficacy (COVID-19) t2	0.23 (0.08)^*^	0.15 (0.10)	0.21 (0.11)	0.25 (0.08)^**^	0.25 (0.08)^**^
Similarity of action t2	0.07 (0.07)	0.08 (0.07)	−0.04 (0.08)	0.12 (0.07)	−0.03 (0.09)
Interaction efficacy (COVID-19) × similarity of action	−0.017 (0.08)	0.23 (0.11)^*^	0.18 (0.14)	−0.06 (0.07)	0.13 (0.10)
*R^2^*	0.40	0.48	0.45	0.49	0.35
*F* (df1 = 4/df2 = 108)	18.17^**^	25.21^**^	22.25^**^	25.69^**^	14.25^**^

* p < 0.05,

** p < 0.01. n = 113. Unstandardized regression coefficients b with standard errors (SE) in parentheses.

We first present the results pertaining to the transfer of efficacy beliefs from the pandemic to the climate crisis. As in Study 1, we find a unique, statistically significant direct transfer effect of COVID-19-related self-efficacy onto its climate-related self-efficacy counterpart (*b* = 0.19^*^ and *b* = 0.23^*^, in Tables 3, 4, respectively), above and beyond the stability of climate-related efficacy between t1 and t2. The same unique direct transfer effect between the two crises is found for positive (*b* = 0.29/0.25^**^) and negative efficacy affect (*b* = 0.24/0.25^**^). Unlike in study 1, however, direct transfer of participatory efficacy is not statistically confirmed (it is fully moderated by similarity of action though; see below). Direct transfer is also not statistically significant in collective efficacy. Throughout, all five climate-related efficacy beliefs show high stability over the course of three months from t1 to t2. Both stability and direct transfer effects are fairly constant in size across the various efficacy beliefs; direct transfer amounts to about a third to a half of the stability effects (coefficients ranging ca. *b* = 0.50 to *b* = 0.60 in stability; ca. *b* = 0.20 to *b* = 0.25 in direct transfer).

Second, we are interested in the role of perceived similarity between the two crises. As shown in Table 3, similarity of threat is positively associated with self-efficacy (*b* = 0.16^*^), participatory efficacy (*b* = 0.31^*^) and positive efficacy affect (*b* = 0.22^*^). This effect tends to be slightly weaker than the direct transfer of efficacy beliefs. Contrary to assumptions, no statistically significant interaction of similarity of threat and COVID-related efficacy beliefs on the climate-related counterparts is found. As shown in Table 4, similarity of action is not associated with any climate-related efficacy beliefs; however, similarity of action moderates the transfer of participatory efficacy (interaction *b* = 0.23^*^). Here, for each step of similarity of action on the five-step response scale, the effect of COVID-19-related participatory efficacy on climate-related participatory efficacy at t2 is additionally increased by.23 steps. The unique simple effect of COVID-19-related participatory efficacy is not statistically significant (*b* = 0.15^n.s.^); thus, the influence of COVID-19-related participatory efficacy fully depends on similarity of action. This indicates that the learning of participatory efficacy beliefs requires a perceived similar context of the COVID-19 and the climate crisis.

### Discussion

Despite its small sample size, and in line with our first research question, Study 2 confirms the transfer of self-efficacy beliefs from the COVID-19 to the climate crisis found in Study 1, and additionally finds transfer of efficacy affect between both crises. Contrary to Study 1, however, the transfer assumption of the first research question was neither supported for participatory efficacy, nor for collective efficacy. When comparing the sizes of the unstandardized regression coefficients across all results of Study 2, the direct impacts of COVID-19-related efficacy and similarity are about a third to a half of the stability of climate-related efficacy. This suggests that efficacy beliefs are indeed changeable and fluid – presumably because the exceptional disruption of the national lockdown provided almost daily action-oriented feedback on the perceived capability of coping with an existential crisis. Regarding our second research question, similarity as a favorable condition for the transfer of efficacy beliefs is only confirmed as a significant interaction between similarity of action and participatory efficacy.

Study 2 comes with important methodological caveats: The findings only apply to Austrian high school graduates and need to be replicated for other countries and other populations. The small sample lacks statistical power to confirm more subtle transfer or moderator effects. Efficacy beliefs were assessed with regard to the overarching goals of climate protection or tackling the COVID-19 crisis; this may attenuate the relevance of the results for specific everyday actions (Lauren et al., 2019). Similarity of threat and similarity of action are derived *post-hoc* from exploratory factor analysis and need not generalize to the communalities between other contexts where transfer may occur. Finally, participatory and collective efficacy named other (young) people as in-group, but the social identity of belonging to this group was not emphasized to the respondents before measuring those efficacy beliefs. Thus, lack of salient group identification could be an alternative explanation for the absent transfer of participatory and collective efficacy from COVID-19 to the climate crisis.

## General discussion

Using the research opportunity to collect survey data when the first COVID-19 lockdown ended in Switzerland and Austria, the present paper explores the transfer of efficacy beliefs from the COVID-19 pandemic to the climate crisis. We find empirical support for our first research question, that COVID-19-related efficacy beliefs directly affect their respective climate-related counterparts, even when controlling for climate-related self-identity or the stability of climate-related efficacy beliefs over time. Our results from samples of two different countries thus support corresponding theoretical assumptions expressed at the beginning of the pandemic (Reese et al., 2020; Lehmann et al., 2021), and extend previous empirical evidence on possible efficacy transfer (Lucarelli et al., 2020; Meijers et al., 2021).

Our results are less clear regarding our second research question on the mechanisms, conditions, and behavioral effects of this efficacy transfer. We find evidence of partial mediation of the effect of COVID-19-related efficacy on private behavior and policy support *via* climate-related efficacy, and of a moderator effect of perceived similarity. These findings do not emerge consistently across the two samples and five efficacy beliefs tested, however. We therefore return to the processes outlined in the introductory sections of this paper on how a transfer from the COVID-19 pandemic to the climate crisis might unfold.

First, the *efficacy transfer process* from the COVID-19 pandemic to the climate crisis appears more robust for self-centered efficacy beliefs, that is to say, self-efficacy and efficacy affect. Transfer of self-efficacy is confirmed in both Study 1 and Study 2; Study 2 additionally finds transfer of positive and negative efficacy affect. By contrast, group-centered beliefs, in other words, participatory and collective efficacy, only turn out to be significant in Study 1. This lack of participatory and collective efficacy transfer may trace back to the absence of collective activities due to the very nature of the lockdown, which prevented students from experiencing collective functioning with their peers (apart from virtual interaction). By contrast, the positive association between COVID-19- and climate-related participatory efficacy in Study 1 may stem from its mostly adult sample who had more opportunities for experiencing successful (that is, pandemic-preventing) collaboration, for example, at their workplace or when caring for their elderly relatives. If, however, the restriction of efficacy transfer to self-centered beliefs as observed in Study 2 is confirmed in future studies using larger, more representative samples, this contradicts Reese et al.'s (2020) optimistic expectation that group processes during the COVID-19 crisis might inform collective appraisal and action regarding the climate crisis.

Second, the effects of COVID-19-related efficacy beliefs on climate-related behavior responses in Study 1 are partially, but not fully mediated by climate-related efficacy beliefs. Thus, our results support previous research on efficacy beliefs as spillover mechanism (e.g., Lauren et al., 2016; Nilsson et al., 2017; Carrico, 2021). It seems that efficacy beliefs play an additional, directly predicting role besides functioning as an indirect, mediating mechanism. Possibly, the unique direct effect on private behavior and policy support stands for a *consistency process* of people striving to uphold a self-image as a conscientious citizen who cares for societal problems. Future studies should thus control for confounding factors which may govern or even distort the relationship between efficacy beliefs and behaviors. Possible confounding factors are, for example, a strong identification with all humanity (Reese et al., 2015), or a strong internal locus of control (Fielding and Head, 2012; Engqvist Jonsson and Nilsson, 2014), but might also encompass attitudinal or trait characteristics, such as e.g., trust in science or high optimism, that might explain both, climate- and COVID-19-related efficacy beliefs. Future studies should include and control for such potential confounding variables. Finally, our results suggest that self-identity also acts as a confounder and needs to be considered in future studies. The exact interplay of self-identity and self-efficacy could not be elucidated in detail in this study. In our view, it should therefore definitely be the subject of future studies.

Third, a perceived similarity of threat between the two crises does not moderate but directly predicts climate-related efficacy beliefs in Study 2. Perceiving both crises similarly as threats seems to advance a *process of raising awareness of the personal role* in managing the climate crisis. Realizing both COVID-19 and climate as pressing crises that call for extensive collaborative efforts increases climate-related self-efficacy, a positive outlook of feeling hopeful and motivated, and, most pronounced, participatory efficacy. This resonates with previous research that perceiving the climate crisis an urgent and important issue increases self-efficacy (Tabernero and Hernández, 2011) and that the COVID-19 pandemic and the climate crisis are perceived as similar threats (Bostrom et al., 2020; Geiger et al., 2021b). Consistent with a potential awareness-raising function of similarity of threat, we do not find any significant influence of similarity of action on efficacy beliefs: Similarity of action refers to short-term and governmental intervention and so does not highlight the role oneself could or should play.

Fourth, and finally, the interaction effect of similarity of action on participatory efficacy, as found in Study 2, points to an efficacy-building *process of learning from similar contexts*. When recognizing as a communality between both crises that state action yields short-term remedies, confidence in one's incremental yet crucial contribution as a member of the collective is transferred from the COVID-19 to the climate context. Lucarelli et al. (2020) report a similar process where considering the COVID-19 and the climate crisis as interdependent builds self-efficacy; however, we find a statistically significant interaction term only for participatory efficacy, in sharp contrast to efficacy theory that underscores learning and applying skills to other, similar contexts as the principal avenue for developing various efficacy beliefs (Bandura, 1977). It seems that learning from similar contexts plays a marginal role in building efficacy beliefs compared to the processes of transfer and awareness-raising, as described above. Presumably, the COVID-19 crisis rather activated or made more salient pre-existing climate efficacy beliefs instead of actual learning and formation of new beliefs taking place.

The *ad hoc* data collection in spring of 2020 was, on the one hand, an exceptional research opportunity but is, on the other hand, a central limitation of the present study. As the pandemic has since progressed through subsequent infection waves triggering repeated lockdowns, the results only offer a snapshot from the initial phase of the pandemic. Possibly, as individuals and collectives struggle(d) in coming to terms with a persistent crisis during the later and current phases, the transfer of efficacy beliefs may have changed because people had varying experiences of mastery and performance accomplishment. It also remains a topic for future research whether transfer from one crisis to the other occurs not just within the same efficacy belief (e.g., from COVID-19- to climate-related self-efficacy, as investigated in the present study), but also between different efficacy beliefs (e.g., from COVID-19-related self-efficacy to climate-related collective efficacy). Both aspects require longitudinal data with larger samples and more survey waves than available for this paper. When Study 2 participants were contacted a third time in April 2021, the sample size dropped to just *n* = 68 respondents which is why we did not include a third measurement point t3 in our longitudinal analysis.

## Conclusions

When the COVID-19 pandemic forced governments to implement swift and radical measures, many voices argued that this might provide a blueprint and door opener for ambitious climate action once the pandemic declined (e.g., Reese et al., 2020; Lehmann et al., 2021). Developing an individual and collective sense of capability to achieve a common goal, be it overcoming the pandemic or reaching the 1.5°C climate target, could be one of the many possible lessons humanity may take from COVID-19. Our results suggest that if the lockdown in the spring of 2020 was experienced as a successful strategy of crisis management, efficacy beliefs for combating climate change increased. Efficacy beliefs are hard to increase in laboratory environments (Hamann and Reese, 2020), and it seems to take substantial interventions to change ingrained beliefs such as a coaching weekend program (Hamann et al., 2021) or even a pandemic lockdown. The regression coefficients observed in our studies are rather small in magnitude, though, so we do not expect massive turnarounds in consumer lifestyles and climate policy acceptance. Moreover, most transfer effects found encompass self-centered efficacy beliefs, so we assume that the pandemic triggers individual rather than collective climate action. Last but not least, it should be kept in mind that transfer functions in both ways – experiences of success may carry over to other contexts as well as experiences of failure, as indicated by the positive sign of our regression coefficients.

Since our data collection after the first COVID-19 infection wave, the world has seen several successive waves. The initially optimistic public debate was replaced by public criticism of restrictive governmental measures. Social trust and public acceptance of the measures have declined (Siegrist and Bearth, 2021). It became apparent that the GHG reductions resulting from the pandemic only marginally contribute to climate targets (Meles et al., 2020). These positive effects on the climate went along with massive costs for welfare and the economy (Elliott et al., 2020; Foad et al., 2021). We may only speculate how individual and societal efficacy transfer processes from COVID-19 have been continuing beyond the timeframe of our fieldwork. Monitoring the development with longitudinal studies would be indispensable to obtain greater clarity. Several parallel processes might have been shaping how efficacy beliefs evolve(d) over the course of the pandemic: A (further) decrease in public acceptance of radical governmental measures could undermine perceived collective efficacy to combat climate change. As the immediate COVID-19 threat fades into the background, the experiences and lessons gained from the pandemic might, as well, and climate-related efficacy beliefs could fall back to pre-pandemic levels. Or, contrariwise, as COVID-19 becomes a less pressing concern, citizens might (again) become more willing to engage with comprehensive state action for protecting the climate, following the finite pool of worries approach (Evensen et al., 2021).

It already seems undisputed that the radical measures taken in the pandemic cannot serve as a blueprint for dealing with the climate crisis; the social and economic costs have been far too high. A careful reappraisal and evaluation of our coping with the COVID-19 crisis in order to draw appropriate lessons are imperative (Howarth et al., 2020). As our results suggest, however, emphasizing the successful management of the COVID-19 crisis will most likely build public confidence that we can combat other global crises, such as the climate crisis, as well.

## Data availability statement

The raw data supporting the conclusions of this article will be made available by the authors, without unduereservation.

## Ethics statement

Ethical review and approval was not required for the study on human participants in accordance with the local legislation and institutional requirements. Written informed consent from the participants' legal guardian/next of kin was not required to participate in this study in accordance with the national legislation and the institutional requirements.

## Author contributions

All authors listed have made a substantial, direct, and intellectual contribution to the work and approved it for publication.

## Funding

This work was supported by the Mercator Foundation Switzerland (Grant Number 2020-416) and was carried out within the Austrian Climate Research Program (Funding No. B960259). Open access funding provided by University of Bern.

## Conflict of interest

Author SS was employed by Joanneum Research Forschungsgesellschaft mbh. The remaining author declares that the research was conducted in the absence of any commercial or financial relationships that could be construed as a potential conflict of interest.

## Publisher's note

All claims expressed in this article are solely those of the authors and do not necessarily represent those of their affiliated organizations, or those of the publisher, the editors and the reviewers. Any product that may be evaluated in this article, or claim that may be made by its manufacturer, is not guaranteed or endorsed by the publisher.
