# Supplementary material for: Has the COVID-19 pandemic strengthened confidence in managing the climate crisis? Transfer of efficacy beliefs after experiencing lockdowns in Switzerland and Austria
Source: Front Psychol. 2022 Oct 10;13:892735. doi: 10.3389/fpsyg.2022.892735 (PMC9589406; doi:10.3389/fpsyg.2022.892735)
Supplement: Supplementary file 1 [file Data_Sheet_1.PDF]

Supplementary Material

Has the COVID19 pandemic strengthened confidence in managing the climate crisis? Transfer of efficacy beliefs after experiencing lockdowns in Switzerland and Austria

Table S.1. Item wordings, descriptive statistics, and scale reliabilities of Study 1

Factor	Item	*M*	*SD*	r_{it}	*α*
Climate-related self-efficacy	I am able to do something for climate protection on my own, even if it is stressful and difficult. #	3.84	.97	.76	.86
	I am confident that I can do something for climate protection on my own. #	3.66	1.05	.76	
COVID19-related self-efficacy	I am able to do something for tackling the COVID19 crisis on my own, even if it is stressful and difficult. #	3.42	1.06	.66	.80
	I am confident that I can do something for tackling the COVID19 crisis on my own. #	3.46	1.04	.66	
Climate-related related participatory efficacy	I am capable of making a small but important contribution to improving climate protection together with other people. #	3.46	1.14	.60	.75
	My participation is an important contribution so that people together advance climate protection. #	2.59	1.24	.60	
COVID19- related participatory efficacy	I am capable of making a small but important contribution to improving tackling the COVID19 crisis together with other people. #	3.56	1.06	.67	.80
	My participation is an important contribution so that people together advance tackling the COVID19 crisis. #	3.48	1.15	.67	

Table S.1. Item wordings, descriptive statistics, and scale reliabilities of Study 1 (continued)

Policy Support	The COVID19 crisis is an opportunity for a greener, more climate-friendly economy.	3.38	1.21	.65	.82
	The COVID19 crisis demonstrates that mitigation of global warming is possible.	3.63	1.21	.68	
	To protect the climate, I would welcome profound governmental measures similar to those taken to combat the COVID19 pandemic.	2.93	1.36	.65	
	Combating climate change requires far-reaching governmental measures.	3.93	1.17	.58	
Private behavior	I gave away or swapped items I no longer needed.	2.10	1.30	.42	.71
	I have repaired items that were broken.	2.47	1.33	.48	
	I bought food from the local area (e.g., neighborhood stores, farms).	3.34	1.34	.37	
	I have canned / made my own food.	2.58	1.46	.37	
	I spent a lot of time in nature.	3.66	1.22	.37	
	I more consciously noticed my surrounding.	3.48	1.18	.45	
	I have decluttered my apartment / house.	2.93	1.41	.48	
Climate-related Self-identity	I am the type of person who acts in a climate-friendly way.	3.64	.87	.86	.92
	Acting climate-friendly is an important part of who I am.	3.45	1.04	.84	
	I see myself as a climate-friendly person.	3.51	.97	.82	

Answer categories for items of private behavior ranged from 1 = ‘not true at all’ to 5 = ‘fully true.’ Answer categories for all other items ranged from 1 = ‘strongly disagree’ to 5 = ‘strongly agree.’ *M* = Mean. *SD* = Standard deviation. r_{it} = Item-total correlation. α = Cronbach’s Alpha. All items were posed in German and translated for this paper. # Item identically phrased as in Study 2.

Table S.2. Intercorrelations between efficacy beliefs in Study 1

	1	2	3
1 Self-Efficacy (COVID19)			
2 Self-Efficacy (Climate)	.34		
3 Participatory Efficacy (COVID19)	.55	.29	
4 Participatory Efficacy (Climate)	.34	.60	.40

Table gives Pearson Correlation Coefficients. All coefficients are (two-tailed) significant with $p<.01$

Table S.3. Model coefficients for the mediation model assuming an indirect effect of COVID19-related self-efficacy on behavioral responses via climate-related self-efficacy, without self-identity as covariate.

	Self-Efficacy (Climate)					Policy Support					Private Behavior				
**Predictor**	**Parameter**	**Estimate**	***SE***	***p***	**95% C.I. (LL, UL)**	**Parameter**	**Estimate**	***SE***	***p***	**95% C.I. (LL, UL)**	**Parameter**	**Estimate**	***SE***	***p***	**95% C.I. (LL, UL)**
Constant	i_M	2.64	.12	.000	(2.41, 2.87)	iy	1.39	.14	.000	(1.11, 1.167)	iy	1.86	.12	.000	(1.63, 2.09)
Self-Efficacy (COVID19)	*a*	.33	.03	.000	(.26, .39)	c'	.09	.03	.006	(.03, .15)	c'	.13	.03	.000	(.07, .18)
Self-Efficacy (Climate)		-	-	-	-	b	.47	.03	.000	(.41, .54)	b	.17	.03	.000	(.12, .23)
	$R^2 = .11$<br>$F(1,989) = 106.80$, $p < .001$<br>$N = 991$					$R^2 = .23$<br>$F(2,988) = 138.49$, $p < .001$<br>$N = 991$					$R^2 = .09$<br>$F(2,991) = 45.33$, $p < .001$<br>$N = 994$				

Notes. Parameters correspond to the paths in Figure 1. Estimate = unstandardized regression coefficient. *SE* = robust standard error (HC3), *95% C.I.* (LL, UL) = 95% bootstrap confidence interval (lower level, upper level).

For policy support the indirect effect of COVID19-related self-efficacy via climate-related self-efficacy results from the product of the parameters $a*b = .33*.47 = .15$ and is statistically significant (*SE(HC3)* = .02, $Z = 8.37$, $p = .000$, 95% C.I.[.12, .19]) according to Sobel's test. The total effect of COVID19-related self-efficacy on policy support amounts to $c = c'+a*b = .24$ ($p = .000$, 95% C.I. [.18, .31]). Thus, the influence of COVID19-related self-efficacy on policy support is partially mediated by climate-related self-efficacy.

For private behavior the indirect effect of COVID19-related self-efficacy via climate-related self-efficacy results from the product of the parameters $a*b = .33*.17 = .06$ and is statistically significant (*SE(HC3)* = .01, $Z = 5.31$, $p = .000$, 95% C.I.[.04, .08]) according to Sobel's test. The total effect of COVID19-related self-efficacy on private behavior amounts to $c = c'+a*b = .18$ ($p = .000$, 95% C.I. [.13, .23]). Thus, the influence of COVID19-related self-efficacy on private behavior is partially mediated by climate-related self-efficacy.

Table S.4. Model coefficients for the mediation model assuming an indirect effect of COVID19-related participatory efficacy on behavioral responses via climate-related participatory efficacy, without self-identity as covariate.

	Participatory Efficacy (Climate)					Policy Support					Private Behavior				
**Predictor**	**Parameter**	**Estimate**	***SE***	***p***	**95% C.I. (LL, UL)**	**Parameter**	**Estimate**	***SE***	***p***	**95% C.I. (LL, UL)**	**Parameter**	**Estimate**	***SE***	***p***	**95% C.I. (LL, UL)**
Constant	i_M	1.53	.12	.000	(1.29, 1.78)	iy	1.91	.12	.000	(1.68, 2.14)	i_y	2.01	.10	.000	(1.82, 2.19)
Participatory Efficacy (COVID19)	*a*	.43	.03	.000	(.36, .49)	c’	.07	.03	.017	(.01, .13)	*c’*	.13	.03	.000	(.08. .19)
Participatory Efficacy (Climate)	-	-	-	-	-	b	.43	.03	.000	(.37, .49)	*b*	.15	.03	.000	(.10, .20)
	$R^2 = .16$<br>$F(1,984) = 154.94$, $p < .001$<br>$N = 986$					$R^2 = .25$<br>$F(2,983) = 152.88$, $p < .001$<br>$N = 986$					$R^2 = .10$<br>$F(2,985) = 52.76$, $p < .001$<br>$N = 988$				

Notes. Parameters correspond to the paths in Figure 1. Estimate = unstandardized regression coefficient. *SE* = robust standard error (HC3), *95% C.I.* (LL, UL) = 95% bootstrap confidence interval (lower level, upper level).

For policy support the indirect effect of COVID19-related participatory-efficacy via climate-related participatory-efficacy results from the product of the parameters $a*b = .43*.43 = .18$ and is statistically significant (*SE(HC3)* = .02, $Z = 9.60$, $p = .000$, 95% C.I.[.15, .22]) according to Sobel's test. The total effect of COVID19-related self-efficacy on policy support amounts to $c = c'+a*b = .26$ ($p = .000$, 95% C.I. [.19, .32]). Thus, the influence of COVID19-related self-efficacy on policy support is partially mediated by climate-related self-efficacy.

For private behavior the indirect effect of COVID19-related participatory-efficacy via climate-related participatory-efficacy results from the product of the parameters $a*b = .43*.15 = .07$ and is statistically significant (*SE(HC3)* = .01, $Z = 5.44$, $p = .000$, 95% C.I.[.04, .09]) according to Sobel's test. The total effect of COVID19-related self-efficacy on private behavior amounts to $c = c'+a*b = .20$ ($p = .000$, 95% C.I. [.15, .25]). Thus, the influence of COVID19-related self-efficacy on private behavior is partially mediated by climate-related self-efficacy.

Table S.5. Check for sample dropout: comparison of socio-demographic distribution in Study 2

**t1 subsample**	***n***	**Gender**		**Age (years)**						**School type**
		Female	Other	16	17	18	19	20	21	General
All	300	41.8	1.4	0.3	28.2	40.8	26.2	3.7	0.7	59.7
Participated in t2	113	53.1	0.0	0.9	33.6	44.2	19.5	1.8	0.0	73.5
No contact data given at t1	69	36.5	3.2	0.0	11.1	47.6	39.7	1.6	0.0	37.7
Contact data given at t1, did not participate in t2	118	33.9	1.7	0.0	32.2	33.9	25.4	6.8	1.7	59.3

All = all wave 1 respondents.

Participated in t2 = wave 1 respondents who gave their contact data and did participate in wave 2, i.e. the sample used for longitudinal analysis.

No contact data given at t1 = wave 1 respondents who did not give their contact data and could not be approached for wave 2, i.e. sample dropout in wave 1.

Contact data given at t1, did not participate in t2 = wave 1 respondents who gave their contact data but did not participate in wave 2, i.e. sample dropout in wave 2.

All values in valid percent (except n). Reference category to gender: male. Reference category to school type: vocational.

Table S.6. Item wordings, descriptive statistics, and scale reliabilities in Study 2

Factor	Item	Climate t1				Climate t2				COVID19 t2			
		M	***SD***	***r_{it}***	*α*	***M***	***SD***	***r_{it}***	*α*	***M***	***SD***	***r_{it}***	*α*
Self-efficacy	I am capable of successfully doing something for [climate protection / tackling the corona crisis].	3.06	0.88	.48	.68	3.29	0.87	.55	.74	4.09	0.95	.51	.74
	I am able to do something for [climate protection / tackling the corona crisis] on my own, even if it is stressful and difficult. #	3.63	.98	.42		3.95	1.01	.58		3.92	1.02	.57	
	I am confident that I can do something for [climate protection / tackling the corona crisis] on my own. #	3.48	1.00	.58		3.62	1.06	.58		3.88	0.99	.62	
Participatory efficacy	I can contribute to achieving improvements in [climate protection / tackling the corona crisis] together with other [young] people.	3.80	1.01	.73	.79	3.49	1.05	.62	.75	4.35	0.74	.58	.73
	I am capable of making a small but important contribution to improving [climate protection / tackling the corona crisis] together with other [young] people. #	3.86	1.07	.62		4.03	1.00	.62		4.37	0.79	.59	
	My participation is an important contribution so that [young] people together advance [climate protection / tackling the corona crisis]. #	3.01	1.16	.57		3.28	1.18	.51		4.04	0.85	.51	
Collective efficacy	Through collaboration, other [young] people and I are capable of [improving climate protection / tackling the corona crisis].	3.86	1.00	.68	.85	3.83	1.03	.77	.89	4.42	0.81	.36	.62
	Through the collective efforts of me and other [young] people, we can achieve progress in [climate protection / tackling the corona crisis].	4.08	1.02	.76		3.88	1.08	.84		4.49	0.75	.53	
	Joint efforts of other [young] people and me can advance [climate protection / tackling the corona crisis], even if we meet resistance or difficulties.	3.68	1.04	.72		3.73	0.94	.76		4.07	0.87	.40	

Table S.6. Item wordings, descriptive statistics, and scale reliabilities in Study 2 (continued)

Positive Efficacy Affect	When acting for [climate protection / tackling the corona crisis], I feel … … hopeful	2.92	1.08	.54	.70	2.96	1.01	.50	.67	3.75	0.90	.39	.55
	… motivated	3.17	1.03	.54		3.26	1.04	.50		3.41	1.02	.39	
Negative Efficacy Affect	… helpless	2.62	1.23	.60	.75	2.67	1.14	.52	.68	2.15	1.06	.58	.73
	… frustrated	2.79	1.26	.60		2.76	1.21	.52		2.48	1.22	.58	
Similarity of threat	How similar do you consider the corona crisis compared to the climate crisis regarding … … the need for timely precautions									0.64	1.36	.40	.60
	… the uncertainty on the best solution									0.19	1.17	.37	
	… the threat to other people									0.96	1.23	.44	
	… the need for collaboration of all people									1.72	0.73	.37	
Similarity of action	… the effectiveness of short-term solutions									-0.15	1.21	.34	.52
	… the public acceptance for extensive governmental measures									-0.46	1.23	.33	
	… the controllability									-0.17	1.20	.34	

Five-step response scales with endpoints labeled 1 = fully disagree and 5 = fully agree (efficacy beliefs) or -2 = very dissimilar and +2 = very similar (similarity). t1 = first survey wave, t2 = second survey wave. *M* = Mean. *SD* = Standard deviation. r_{it} = Item-total correlation. α = Cronbach's Alpha. All items were posed in German and translated for this paper. # Item identically phrased as in Study 1.

Table S.7. Factor analysis of similarity attributes

**Item**	**Factor threat**	**Factor action**	**Factor other**
How similar do you consider the corona crisis compared to the climate crisis regarding …	.60		
… the need for timely precautions			
… the uncertainty on the best solution	.44		.59
… the threat to other people	.62	.26	.23
… the need for collaboration of all people	.77		
… the effectiveness of short-term solutions		.62	.25
… the public acceptance for extensive governmental measures		.74	
… the controllability		.72	
… the threat to the economy #			.75
… the threat to you #	.38		.22
… the importance of expert knowledge #	.49	.21	-.50
Eigenvalue	2.36	1.38	1.24
Explained variance	23.6%	13.8%	12.4%

Principal component analysis with varimax rotation. Table gives factor loadings, coefficients < .20 omitted. # Item not included in similarity factor indices.

Table S.8. Intercorrelations between mean factor indices of efficacy beliefs in Study 2

**Factor**	**Climate**					**COVID19**				
	**SE**	**PE**	**CE**	**EA+**	**EA-**	**SE**	**PE**	**CE**	**EA+**	**EA-**
Self-efficacy (SE)	1	.60	.51	.51	-.01	1				
Participatory efficacy (PE)	.68	1	.79	.57	.06	.70	1			
Collective efficacy (CE)	.55	.80	1	.55	.01	.63	.73	1		
Positive efficacy affect (EA+)	.59	.57	.47	1	-.03	.34	.35	.32	1	
Negative efficacy affect (EA-)	-.13	-.17	-.07	-.18	1	-.06	-.01	-.10	-.28	1

Table gives Pearson Correlation Coefficients. Coefficients $r>|.27|$ are (two-tailed) significant with $p<.01$. Climate: triangle above the main diagonal gives t1, triangle below the main diagonal gives t2.
